# Supplementary material for: Appropriateness of high-priority criteria and safety of endoscopy procedures during the COVID-19 lockdown
Source: PLoS One. 2022 Apr 28;17(4):e0267112. doi: 10.1371/journal.pone.0267112 (PMC9049498; doi:10.1371/journal.pone.0267112)
Supplement: S1 Fig — COVID-19 infected endoscopists (*) and number of endoscopy procedures by all endoscopists during the pre-lockdown cohort (A) and lockdown cohort (B). (DOCX) [file pone.0267112.s001.docx]

**S1 Fig. COVID-19 infected endoscopists (*) and number of endoscopy procedures by all endoscopists during the pre-lockdown cohort (1A) and lockdown cohort (1B).**

**1A**

**
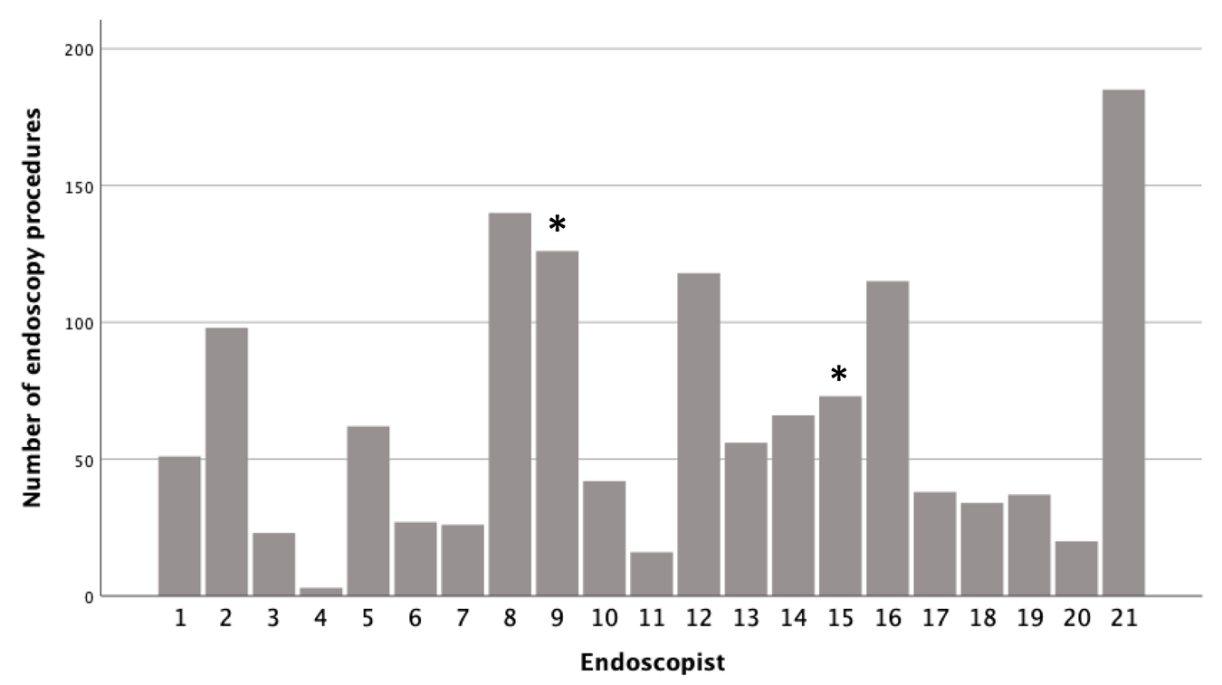
**

**1B**

**
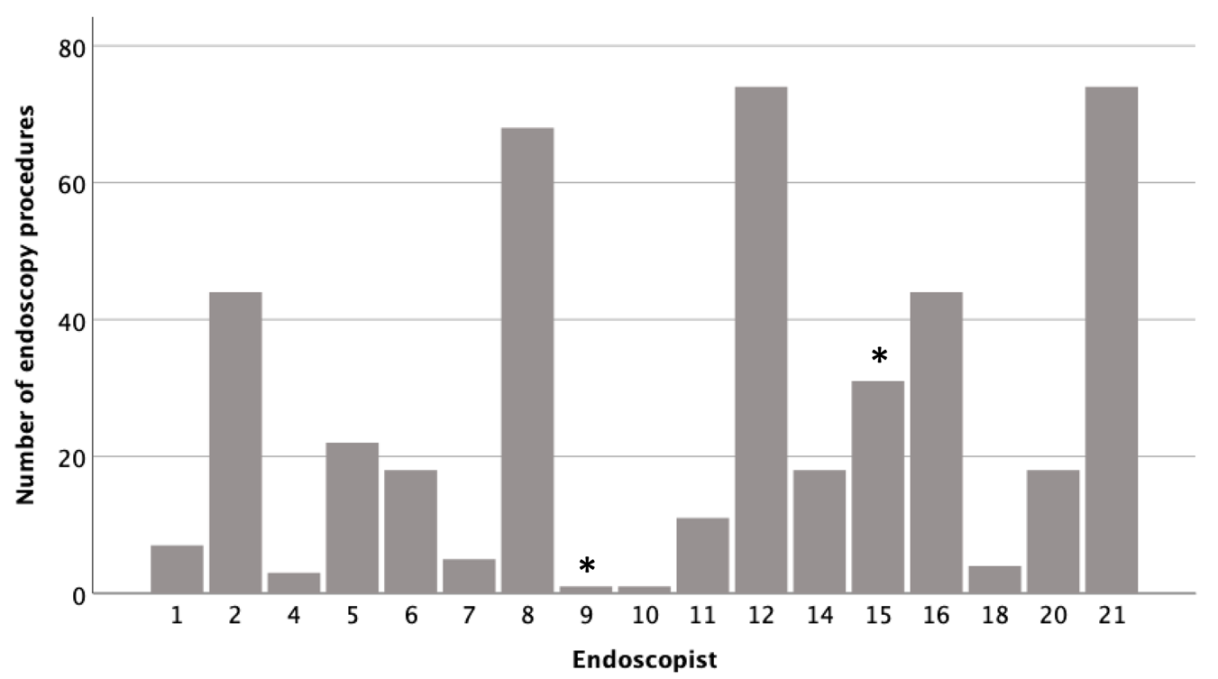
**
