# Supplementary material for: Appropriateness of high-priority criteria and safety of endoscopy procedures during the COVID-19 lockdown
Source: PLoS One. 2022 Apr 28;17(4):e0267112. doi: 10.1371/journal.pone.0267112 (PMC9049498; doi:10.1371/journal.pone.0267112)
Supplement: S1 Table — (DOCX) [file pone.0267112.s002.docx]

**S1 Table. Recommendations for gastrointestinal procedures during the COVID-19 pandemic and protective measures adopted by our endoscopy unit.**

|  | Own  endoscopy unit | SEPD/AEG/  SEED (statement on 18^th^ March) | ESGE (statement released on 18^th^ March, full on 17^th^ April) | AGA (released on 15^th^ March) |
| --- | --- | --- | --- | --- |
| General measures | Yes | Yes | Yes | Yes |
| Measures related to the procedure | | | | |
| - Risk stratification | Yes | Yes | Yes | Yes |
| - Team approach for decision making for difficult cases | Yes | Yes | - | Yes |
| - Telephone call to the patient prior to endoscopy | Yes | Yes | Yes | - |
| - Telephone consultation with the referring provider | No | - | - | Yes |
| Measures related to the patient | | | | |
| - Surgical mask | Yes | Yes | Yes | Yes |
| - Hands wash (alcohol-based solution, gloves…) | Yes | Yes | - | Yes |
| - Telehealth consultation prior to appointment | Yes | Yes | Yes | Yes |
| - Point-of-care test | No |  | Yes (if available) | - |
| - Epidemiological and clinical questionnaire triage | Yes | Yes | Yes | - |
| - On-site temperature measurement | Yes | Yes | Yes | - |
| - Cleaning room protocol | Yes | Yes | Yes | - |
| - Cleaning procedure rooms | No* | Yes | Yes | - |
| Measures related to staff prevention | | | | |
| - Triage for symptoms and signs for COVID-19 | Yes | Yes | Yes | - |
| - Surgical mask | Yes | Yes | Yes | Yes |
| - Hand wash | Yes | Yes | Yes | Yes |
| - Respirator mask (FPP2, FPP3) | No* | Yes | Yes | Yes |
| - Eye protection (goggles, face shield) | Yes | Yes | Yes | - |
| - Gloves | Yes (single) | Yes | Yes | Yes (double) |
| - Hairnet | Yes | Yes | Yes | - |
| - Sleeve waterproof gown | Yes | Yes | Yes | - |
| - Shoe protection (covers, booties, …) | Yes | Yes | Yes | - |
| - Disposable pajama | Yes | - | - | - |
| - Full-sleeved gown | No* | No* | Yes | - |
| - Negative pressure room | No | - | No* | Yes |
| - Course on personal protective equipment | Yes | - | Yes | - |
| - Tracking of patients | No | - | Yes | - |
| - Safety protocol for COVID-19 cases | Yes | - | Yes | - |
| - Waste management | Yes | Yes | Yes | - |
| - Postponing procedures in positive COVID-19 cases (when possible) | Yes | Yes | Yes | - |
| Cleaning endoscopic disinfection | | | | |
| - Standard | Yes | Yes | Yes | Yes |
| - Viricida | Yes | Yes | Yes | No |
| Social distancing | | | | |
| - Split teams (shifts, …) | Yes | Yes | - | - |
| - Accompanying individual remain outside the endoscopy unit | Yes | Yes | Yes | - |

* Other than high-risk procedures, suspected or confirmed positive cases were not reported
