# Supplementary material for: Appropriateness of high-priority criteria and safety of endoscopy procedures during the COVID-19 lockdown
Source: PLoS One. 2022 Apr 28;17(4):e0267112. doi: 10.1371/journal.pone.0267112 (PMC9049498; doi:10.1371/journal.pone.0267112)
Supplement: S2 Table — IBD: Inflammatory bowel disease, GAVE: Gastric antral vascular ectasia. (DOCX) [file pone.0267112.s003.docx]

**S2 Table. List of endoscopic findings considered to be relevant.**

| **Relevant endoscopic findings** |
| --- |
| Cancer |
| Advanced neoplasia |
| Active IBD |
| Gastric/duodenal ulcer |
| Angiodysplasia |
| Gastroesophageal varices |
| Barrett’s esophagus |
| Severe esophagitis |
| Foreign body |
| GAVE |
| Erosive gastritis/duodenitis |
| Actinic proctitis |
| Colonic ulcers |
| Subepithelial lesions |
| Biliary obstruction |
| Chronic pancreatitis |
| Choledocolitiasis |
| Others: Eroded gastric polyp, dieulafoy lesion, stenosis and lymph nodes |

IBD: Inflammatory bowel disease, GAVE: Gastric antral vascular ectasia
