# Supplementary material for: Appropriateness of high-priority criteria and safety of endoscopy procedures during the COVID-19 lockdown
Source: PLoS One. 2022 Apr 28;17(4):e0267112. doi: 10.1371/journal.pone.0267112 (PMC9049498; doi:10.1371/journal.pone.0267112)
Supplement: S3 Table — PEG: Percutaneous endoscopic gastrostomy, GI: Gastrointestinal, FOBT: Fecal occult blood test, IBD: Inflammatory bowel disease, IBS: Irritable bowel syndrome. (DOCX) [file pone.0267112.s004.docx]

**S3 Table. Clinical indications categorized according to the ESGE criteria of inpatient endoscopy procedures in the pre-lockdown and lockdown cohorts.**

|  | **Pre-alarm cohort**  **(n=196)** | **Alarm cohort**  **(n=133*)** | **p** |
| --- | --- | --- | --- |
| **High-priority criteria, n (%)** | **155 (79.1%)** | **114 (85.7%)** | **0.146** |
| Therapeutic endoscopy | 1 (0.6%) | 0 |  |
| PEG, nasogastric and nasojejunal tubes | 1 (0.6%) | 1 (0.9%) |  |
| Dysphagia or dyspepsia with alarm symptoms | 3 (1.9%) | 7 (6.1%) |  |
| Upper GI bleeding | 26 (16.8%) | 23 (20.2%) |  |
| Rectal bleeding | 15 (9.7%) | 21 (18.4%) |  |
| Colonoscopy for melena after negative upper GI endoscopy | 1 (0.6%) | 1 (0.9%) |  |
| Severe anemia | 39 (25.2%) | 18 (15.8%) |  |
| Biopsy for pathology assessment | 4 (2.6%) | 0 |  |
| Positive FOBT | 3 (1.9%) | 0 |  |
| Foreign body | 13 (8.4%) | 5 (4.4%) |  |
| Radiologic evidence of mass | 12 (7.7%) | 6 (5.3%) |  |
| Pancreatic mass | 6 (3.9%) | 5 (4.4%) |  |
| Pancreatic or biliary stent | 2 (1.3%) | 3 (2.6%) |  |
| Obstructive jaundice | 29 (18.7%) | 24 (21.1%) |  |
| **Low-priority criteria, n (%)** | **20 (10.2%)** | **3 (2.3%)** | **0.007** |
| Endoscopic variceal ligation | 2 (10%) | 0 |  |
| Iron deficiency anemia | 1 (5%) | 0 |  |
| Achalasia | 3 (15%) | 0 |  |
| Surveillance for Barrett, gastric atrophy and IBD | 7 (35%) | 2 (66.7%) |  |
| IBS-like symptoms | 7 (35%) | 1 (33.3%) |  |
| **Not classifiable, n (%)** | **21 (10.7%)** | **16 (12%)** | **0.725** |

PEG: Percutaneous endoscopic gastrostomy, GI: Gastrointestinal, FOBT: Fecal occult blood test, IBD: Inflammatory bowel disease, IBS: Irritable bowel syndrome

* There were 2 inpatient procedures in the alarm cohort with non-registered indications.
