# Supplementary material for: Appropriateness of high-priority criteria and safety of endoscopy procedures during the COVID-19 lockdown
Source: PLoS One. 2022 Apr 28;17(4):e0267112. doi: 10.1371/journal.pone.0267112 (PMC9049498; doi:10.1371/journal.pone.0267112)
Supplement: S4 Table — A. Clinical indications of outpatient gastrointestinal (GI) upper endoscopy procedures in the pre-lockdown and lockdown cohorts categorized according to ESGE criteria. B. Clinical indications of outpatient GI lower endoscopy procedures in the pre-lockdown and lockdown cohorts categorized according to ESGE criteria. PEG: Percutaneous endoscopic gastrostomy, GI: Gastrointestinal, IBD: Inflammatory bowel disease, IBS: Irritable Bowel Syndrome, FOBT: Fecal occult blood test. (DOCX) [file pone.0267112.s005.docx]

**S4 Table. Table 4A. Clinical indications of outpatient gastrointestinal (GI) upper endoscopy procedures in the pre-lockdown and lockdown cohorts categorized according to ESGE criteria. Table 4B. Clinical indications of outpatient GI lower endoscopy procedures in the pre-lockdown and lockdown cohorts categorized according to ESGE criteria.**

**4A**

|  | **Pre-alarm cohort (n=259)** | **Alarm cohort**  **(n=92)** | **p** |
| --- | --- | --- | --- |
| **High-priority criteria, n (%)** | **117 (45.2%)** | **50 (54.3%)** | **0.145** |
| Therapeutic endoscopy | 0 | 1 (1.1%) | 0.265 |
| PEG | 4 (1.6%) | 0 | 0.577 |
| Dysphagia or dyspepsia with alarm symptoms | 64 (25.1%) | 20 (21.7%) | 0.572 |
| Upper GI bleeding | 4 (1.6%) | 1 (1.1%) | 1 |
| Severe anemia | 15 (5.9%) | 6 (6.5%) | 0.802 |
| Biopsy for pathology assessment | 6 (2.4%) | 5 (5.4%) | 0.169 |
| Radiologic evidence of mass | 11 (4.3%) | 8 (8.7%) | 0.117 |
| Pancreatic mass | 13 (5.1%) | 9 (9.8%) | 0.134 |
| **Low-priority criteria, n (%)** | **96 (37.1%)** | **19 (20.7%)** | **0.004** |
| Endoscopic variceal ligation | 8 (3.1%) | 5 (5.4%) | 0.342 |
| Iron deficiency anemia | 17 (6.7%) | 6 (6.5%) | 1 |
| Achalasia | 1 (0.4%) | 0 | 1 |
| Surveillance for Barrett, gastric atrophy and IBD | 22 (8.6%) | 4 (4.3%) | 0.249 |
| Post-endoscopic resection, surgical resection and post-polypectomy surveillance | 6 (2.4%) | 3 (3.3%) | 0.704 |
| Hereditary syndromes | 7 (2.7%) | 0 | 0.196 |
| IBS-like symptoms | 5 (2%) | 1 (1.1%) | 1 |
| Reflux-disease and dyspepsia without alarm symptoms | 25 (9.8%) | 0 | 0.001 |
| Screening in high-risk patients for cancer | 1 (0.4%) | 0 | 1 |
| **Not classifiable, n (%)** | **46 (17.8%)** | **23 (25%)** | **0.169** |

PEG: Percutaneous endoscopic gastrostomy, GI: Gastrointestinal, IBD: Inflammatory bowel disease, IBS: Irritable Bowel Syndrome

**4B**

|  | **Pre-alarm cohort**  **(n=454)** | **Alarm cohort**  **(n=227)** | **p** |
| --- | --- | --- | --- |
| **High-priority criteria, n (%)** | **208 (45.8%)** | **148 (65.2%)** | **<0.001** |
| Therapeutic endoscopy | 6 (1.3%) | 1 (0.4%) | 0.434 |
| Rectal bleeding | 52 (11.5%) | 21 (9.3%) | 0.432 |
| Colonoscopy for melena after negative upper GI endoscopy | 0 | 1 (0.4%) | 0.334 |
| Severe anemia | 12 (2.6%) | 12 (5.3%) | 0.120 |
| Biopsy for pathology assessment | 4 (0.9%) | 0 | 0.307 |
| Positive FOBT | 119 (26.3%) | 99 (43.6%) | <0.001 |
| Radiologic evidence of mass | 15 (3.5%) | 14 (6.2%) | 0.106 |
| **Low-priority criteria, n (%)** | **230 (50.7%)** | **71 (31.3%)** | **<0.001** |
| Iron deficiency anemia | 22 (4.9%) | 9 (4%) | 0.699 |
| Surveillance for IBD | 38 (8.4%) | 13 (5.7%) | 0.280 |
| Post-endoscopic resection, surgical resection and post-polypectomy surveillance | 118 (26%) | 33 (14.5%) | 0.001 |
| Hereditary syndromes | 8 (1.8%) | 1 (0.4%) | 0.285 |
| IBS-like symptoms | 27 (6%) | 9 (4%) | 0.364 |
| Screening in high-risk patients for cancer | 16 (3.5%) | 6 (2.6%) | 0.649 |
| **Not classifiable, n (%)** | **16 (3.5%)** | **8 (3.5%)** | **1** |

GI: gastrointestinal, FOBT: Fecal occult blood test, IBD: Inflammatory bowel disease, IBS: Irritable Bowel Syndrome
