# Supplementary material for: Appropriateness of high-priority criteria and safety of endoscopy procedures during the COVID-19 lockdown
Source: PLoS One. 2022 Apr 28;17(4):e0267112. doi: 10.1371/journal.pone.0267112 (PMC9049498; doi:10.1371/journal.pone.0267112)
Supplement: S5 Table — IBD: Inflammatory bowel disease, GAVE: Gastric antral vascular ectasia. (DOCX) [file pone.0267112.s006.docx]

**S5 Table. Relevant endoscopic finding in the pre-lockdown and lockdown cohorts.**

|  | **Pre-alarm cohort**  **(n=167)** | **Alarm cohort**  **(n=101)** | **p** |
| --- | --- | --- | --- |
| **Relevant endoscopic findings among high-priority criteria, n (%)** | **86 (51.5%)** | **66 (65.3%)** | **0.031** |
| Cancer | 24 (27.9%) | 21 (31.8%) |  |
| Advanced neoplasia | 38 (44.2%) | 25 (37.9%) |  |
| Active IBD | 0 | 7 (10.6%) |  |
| Gastric/duodenal ulcer | 1 (1.2%) | 0 |  |
| Angiodysplasia | 9 (10.5%) | 5 (7.6%) |  |
| Gastroesophageal varices | 1 (1.2%) | 0 |  |
| Barrett’s esophagus | 1 (1.2%) | 0 |  |
| Severe esophagitis | 2 (2.3%) | 1 (1.5%) |  |
| Foreign body | 0 | 0 |  |
| GAVE | 1 (1.2%) | 1 (1.5%) |  |
| Erosive gastritis/duodenitis | 2 (2.3%) | 3 (4.5%) |  |
| Actinic proctitis | 1 (1.2%) | 0 |  |
| Colonic ulcers | 0 | 1 (1.5%) |  |
| Subepithelial lesions | 4 (4.7%) | 1 (1.5%) |  |
| Biliary obstruction | 0 | 0 |  |
| Chronic pancreatitis | 0 | 1 (1.5%) |  |
| Choledocolitiasis | 0 | 1 (1.5%) |  |
| Others: Eroded gastric polyp, dieulafoy lesion, stenosis and lymph nodes | 2 (2.3%) | 0 |  |
| **Relevant endoscopic findings among low-priority criteria, n (%)** | **67 (40.1%)** | **19 (18.9%)** | **<0.001** |
| Cancer | 3 (4.5%) | 2 (10.5% |  |
| Advanced neoplasia | 15 (22.4%) | 6 (31.6%) |  |
| Active IBD | 11 (16.4%) | 6 (31.6%) |  |
| Gastric/duodenal ulcer | 0 | 0 |  |
| Angiodysplasia | 8 (11.9%) | 2 (10.5%) |  |
| Gastroesophageal varices | 6 (9%) | 1 (5.3%) |  |
| Barrett’s esophagus | 14 (20.9%) | 1 (5.3%) |  |
| Severe esophagitis | 0 | 0 |  |
| Foreign body | 0 | 0 |  |
| GAVE | 1 (1.5%) | 0 |  |
| Erosive gastritis/duodenitis | 6 (9%) | 0 |  |
| Actinic proctitis | 1 (1.5%) | 1 (5.3%) |  |
| Colonic ulcers | 0 | 0 |  |
| Subepithelial lesions | 1 (1.5%) | 0 |  |
| Biliary obstruction | 0 | 0 |  |
| Chronic pancreatitis | 0 | 0 |  |
| Choledocolitiasis | 0 | 0 |  |
| Others: Eroded gastric polyp, dieulafoy lesion, stenosis and lymph nodes | 1 (1.5%) | 0 |  |
| **Relevant endoscopic findings among not classifiable, n (%)** | **14 (8.4%)** | **16 (15.8%)** | **0.073** |
| Cancer | 2 (14.3%) | 2 (14.3%) |  |
| Advanced neoplasia | 0 | 1 (6.3%) |  |
| Active IBD | 1 (7.1%) | 0 |  |
| Gastric/duodenal ulcer | 0 | 0 |  |
| Angiodysplasia | 0 | 0 |  |
| Gastroesophageal varices | 5 (35.7%) | 4 (25%) |  |
| Barrett’s esophagus | 0 | 1 (6.3%) |  |
| Severe esophagitis | 0 | 0 |  |
| Foreign body | 0 | 0 |  |
| GAVE | 0 | 0 |  |
| Erosive gastritis/duodenitis | 3 (21.4%) | 4 (25%) |  |
| Actinic proctitis | 0 | 0 |  |
| Colonic ulcers | 0 | 0 |  |
| Subepithelial lesions | 2 (14.3%) | 0 |  |
| Biliary obstruction | 0 | 0 |  |
| Chronic pancreatitis | 1 (7.1%) | 2 (12.5%) |  |
| Choledocolitiasis | 0 | 0 |  |
| Others: Eroded gastric polyp, dieulafoy lesion, stenosis and lymph nodes | 0 | 1 (6.3%) |  |

IBD: Inflammatory bowel disease, GAVE: Gastric antral vascular ectasia
