# Supplementary material for: Appropriateness of high-priority criteria and safety of endoscopy procedures during the COVID-19 lockdown
Source: PLoS One. 2022 Apr 28;17(4):e0267112. doi: 10.1371/journal.pone.0267112 (PMC9049498; doi:10.1371/journal.pone.0267112)
Supplement: Attachment — Submitted filename: Response to Reviewers.docx [file pone.0267112.s007.docx]

Response to the editor and reviewers

PONE-D-21-12011
Appropriateness of high-priority criteria and safety of endoscopy procedures during the COVID-19 lockdown

PLOS ONE

Journal Requirements:

When submitting your revision, we need you to address these additional requirements.

1. Please ensure that your manuscript meets PLOS ONE's style requirements, including those for file naming. The PLOS ONE style templates can be found at

<https://journals.plos.org/plosone/s/file?id=wjVg/PLOSOne_formatting_sample_main_body.pdf> and <https://journals.plos.org/plosone/s/file?id=ba62/PLOSOne_formatting_sample_title_authors_affiliations.pdf>

**We have modified our manuscript according to PLOS ONE's style requirements.**

2. Please review your reference list to ensure that it is complete and correct. If you have cited papers that have been retracted, please include the rationale for doing so in the manuscript text, or remove these references and replace them with relevant current references. Any changes to the reference list should be mentioned in the rebuttal letter that accompanies your revised manuscript. If you need to cite a retracted article, indicate the article’s retracted status in the References list and also include a citation and full reference for the retraction notice.

**We have reviewed our reference list and we have not made any changes.**

3. PLOS requires an ORCID iD for the corresponding author in Editorial Manager on papers submitted after December 6th, 2016. Please ensure that you have an ORCID iD and that it is validated in Editorial Manager. To do this, go to ‘Update my Information’ (in the upper left-hand corner of the main menu), and click on the Fetch/Validate link next to the ORCID field. This will take you to the ORCID site and allow you to create a new iD or authenticate a pre-existing iD in Editorial Manager. Please see the following video for instructions on linking an ORCID iD to your Editorial Manager account: <https://www.youtube.com/watch?v=_xcclfuvtxQ>
[Note: HTML markup is below. Please do not edit.]

**We have registered the ORCID iD for the corresponding author.**

Reviewers' comments:

Reviewer's Responses to Questions

**Comments to the Author**

1. Is the manuscript technically sound, and do the data support the conclusions?

The manuscript must describe a technically sound piece of scientific research with data that supports the conclusions. Experiments must have been conducted rigorously, with appropriate controls, replication, and sample sizes. The conclusions must be drawn appropriately based on the data presented.

Reviewer #1: Yes

Reviewer #2: Yes

2. Has the statistical analysis been performed appropriately and rigorously?

Reviewer #1: Yes

Reviewer #2: Yes

3. Have the authors made all data underlying the findings in their manuscript fully available?

The [PLOS Data policy](http://www.plosone.org/static/policies.action#sharing) requires authors to make all data underlying the findings described in their manuscript fully available without restriction, with rare exception (please refer to the Data Availability Statement in the manuscript PDF file). The data should be provided as part of the manuscript or its supporting information, or deposited to a public repository. For example, in addition to summary statistics, the data points behind means, medians and variance measures should be available. If there are restrictions on publicly sharing data—e.g. participant privacy or use of data from a third party—those must be specified.

Reviewer #1: Yes

Reviewer #2: Yes

4. Is the manuscript presented in an intelligible fashion and written in standard English?

PLOS ONE does not copyedit accepted manuscripts, so the language in submitted articles must be clear, correct, and unambiguous. Any typographical or grammatical errors should be corrected at revision, so please note any specific errors here.

Reviewer #1: Yes

Reviewer #2: Yes

5. Review Comments to the Author

Please use the space provided to explain your answers to the questions above. You may also include additional comments for the author, including concerns about dual publication, research ethics, or publication ethics. (Please upload your review as an attachment if it exceeds 20,000 characters)

Reviewer #1: This is an interesting study. Although most of the countries were under impact of COVID-19 pandemic situation, some of the area had not suffered the serious condition such as east Asia countries. The study can help those area further management before outbreak and lock down.

**We thank the reviewer for the comment. We agree. Our study showed results with references to the local burden of COVID-19 infection because these specific data allow for comparations and extrapolation of our methods and results to other endoscopy units. We commented this issue in the discussion section (page 23).**

Reviewer #2: I read with great interest this study, aimed at evaluating the appropriateness of endoscopy procedures during the COVID-19 pandemic lockdown as the rate of high-priority procedures according to the ESGE criteria. Furthermore, the authors evaluated the effectiveness (rate of relevant endoscopic findings, advanced neoplasia/cancer and cancer detection), non-scheduled visits to the hospital and patients and endoscopy staff safety.
By comparing 2 different cohort (lockdown cohort vs pre-lockdown cohort), there was a 49.7% reduction in procedures compared to the pre-lockdown cohort. However, there was a significant increase in the percentage of high-priority indications in the lockdown cohort compared to the pre-lockdown cohort. Similarly, a higher percentage of relevant lesions were observed among high-priory indications in the lockdown cohort compared to the pre-lockdown cohort

The study is well written and well conducted, tables and figures are clear and explicative.

I have only minor comments that I believe could improve the manuscript.

**We thank the reviewer for the time to review and the comments to improve the manuscript. We have updated the main text and the references section with the suggested modifications.**

No data have been reported on urgent/emergency endoscopy (i.e. for gastrointestinal bleeding or foreign bodies). In my mind, elective endoscopic activities have been deeply reduced during pandemic, but gastrointestinal urgencies should not be influenced by Covid-19. Do you have data about endoscopy volume in the emergency setting?

**We have added the numbers of urgent procedures in both cohorts in the results section (page 10, figure 2). In addition, we have registered all clinical indications for inpatients/urgent endoscopy in supplementary table 3 (page 11).**

**We agree with the comment of the reviewer and that is why we focused on the evaluation of the appropriateness and safety on outpatient endoscopy procedures due to the usually unavoidable and mandatory urgent indication nature of the procedures in hospitalized patients independently of COVID-19 local burden (discussion section page 19).**

In the Introduction section, I would like that the Authors highlight the impact of Covid-19 p on Gastroenterologists and Endoscopists, considering the high-risk procedures they perform (add the reference: Imperatore N, Rispo A, Lombardi G. The price of being a doctor during the COVID-19 outbreak. Gut. 2020;69:1544-1545. doi: 10.1136/gutjnl-2020-321646).

**We have added this reference in the introduction section (ref 10). Thank you for your input.**

6. PLOS authors have the option to publish the peer review history of their article ([what does this mean?](https://journals.plos.org/plosone/s/editorial-and-peer-review-process#loc-peer-review-history)). If published, this will include your full peer review and any attached files.

If you choose “no”, your identity will remain anonymous but your review may still be made public.

**Do you want your identity to be public for this peer review?** For information about this choice, including consent withdrawal, please see our [Privacy Policy](https://www.plos.org/privacy-policy).

Reviewer #1: No

Reviewer #2: No
